# *Rhus coriaria* (Sumac) Fruit Extract Enhances the Biocompatibility of a Propolis-Based Herbal Formulation for Oral Mucositis: Biochemical and Gene Expression Analyses in Zebrafish Embryos

**DOI:** 10.3390/molecules31132312

**Published:** 2026-07-01

**Authors:** Zeynep Ceren Çelik, İsmail Ünal, Merih Beler, Derya Cansız, Saliha Şahin, Büşra Karkar, Cigdem Elbek Cubukcu, Ebru Emekli-Alturfan

**Affiliations:** 1 Department of Restorative Dentistry, Faculty of Dentistry, Bursa Uludag University, 16120 Bursa, Türkiye; 2 Department of Medical Biochemistry, Faculty of Medicine, Istanbul Medipol University, 34810 Istanbul, Türkiye; 3 Department of Biochemistry, Institute of Health Sciences, Marmara University, 34854 Istanbul, Türkiye; 4 Department of Chemistry, Faculty of Science and Arts, Bursa Uludag University, 16210 Bursa, Türkiye; 5 Department of Pedodontics, Faculty of Dentistry, Bursa Uludag University, 16120 Bursa, Türkiye; 6 Department of Basic Medical Sciences, Faculty of Dentistry, Marmara University, 34854 Istanbul, Türkiye

**Keywords:** *Rhus*, propolis, biocompatibility, zebrafish embryos, oxidative stress, gene expression

## Abstract

We investigated the biocompatibility of a novel oral herbal formulation containing *Rhus coriaria* (sumac) extract plus propolis compared with a propolis-only formulation using zebrafish embryos. Zebrafish embryos (AB/AB strain) were exposed to 3,4-dichloroaniline (DCA), a propolis-only formulation, or a combined sumac–propolis formulation from 24 to 72 h post-fertilization. Biochemical endpoints—including lipid peroxidation (LPO), nitric oxide (NO), acetylcholinesterase (AChE), and superoxide dismutase (SOD)—were evaluated, while gene expression levels of fabp10a, cyp1a, vtg, bax, bcl2, gfap, erg, and igf2 were quantified using RT–PCR. Statistical analysis was performed using one-way ANOVA with a significance threshold of *p* < 0.05. DCA increased oxidative stress (LPO: *p* < 0.001; NO: *p* < 0.01) with reduced SOD (*p* < 0.01). The propolis-only formulation increased LPO (*p* < 0.01) and decreased SOD (*p* < 0.05) versus control, whereas the sumac + propolis formulation showed milder edema, normalized LPO and NO (both *p* < 0.05 vs. DCA), and increased SOD versus control (*p* < 0.05), DCA (*p* < 0.001), and propolis-only (*p* < 0.001); AChE was unchanged (*p* > 0.05). At the transcript level, DCA upregulated fabp10a/cyp1a/vtg (all *p* < 0.0001) and bax (*p* < 0.0001) while downregulating bcl2 (*p* < 0.001), erg (*p* < 0.0001), and igf2 (*p* < 0.01); these alterations were largely mitigated by the sumac + propolis formulation (vtg lower vs. DCA *p* < 0.0001 and vs. propolis-only *p* < 0.001; igf2 higher vs. DCA *p* < 0.001 and vs. propolis-only *p* < 0.01). Overall, incorporating sumac into a propolis-based formulation improved biocompatibility in zebrafish embryos, supporting further evaluation of this strategy for oral mucositis-oriented applications.

## 1. Introduction

Factors leading to changes in the oral mucosa include bacterial, fungal, and viral infections; physical or thermal irritants such as prosthetic use; immune dysregulation; systemic diseases; neoplasms; head and neck radiotherapy and chemotherapy; medications; chemical exposures such as tobacco and alcohol; and mechanical trauma [1,2]. When immune responses are compromised and DNA repair capacity is impaired, oral epithelial atrophy may occur, resulting in delayed healing of oral ulcers. In particular, painful ulcerative lesions induced by head and neck radiotherapy (RT) and chemotherapy (CT), collectively termed oral mucositis (OM), predispose patients to secondary infections, compromise oral hygiene due to pain and limited mouth opening, impair nutrition and quality of life, and increase the risk of dental caries [3].

Current clinical management strategies for OM include supportive care and preventive approaches. Evidence-based guidelines, including those from the Multinational Association of Supportive Care in Cancer/International Society of Oral Oncology (MASCC/ISOO), also highlight the growing interest in natural agents and bioactive products for the prevention and/or treatment of OM [4,5,6]. Among these, honeybee-derived products—particularly propolis and honey—have attracted considerable attention due to their reported antimicrobial, antioxidant, and anti-inflammatory activities and their potential to support mucosal healing [5,6].

Propolis, a resinous material produced by honeybees from plant exudates, is chemically complex and typically rich in phenolic acids, flavonoids, and other bioactive constituents. This compositional diversity is closely linked to its biological activity, yet it also results in variability between propolis samples depending on botanical and geographical origins. Consequently, although propolis has been investigated as a promising adjunctive agent for OM [7], its efficacy and tolerability may differ across individuals and formulations [8]. In some cases, propolis can irritate oral tissues and may trigger or exacerbate hypersensitivity reactions, potentially worsening mucosal symptoms [9]. Therefore, combining propolis with complementary phytochemicals or developing optimized formulations may represent a rational strategy to enhance biocompatibility while maintaining or improving biological activity.

*Rhus coriaria* L. (sumac), widely used in Türkiye as a culinary spice and traditional remedy, has been reported to exhibit antioxidant and antibacterial properties [10]. It is rich in diverse phytochemical classes, including flavonoids, tannins, polyphenolic compounds, and organic acids. Owing to these properties, sumac has been utilized in traditional medicine for wounds, ulcers, and mucosal inflammation. Collectively, the literature suggests that sumac constitutes an attractive source of bioactive compounds that may complement honeybee products such as propolis, particularly in the context of oxidative stress and inflammation-driven mucosal injury.

Oxidative stress plays a central role in the biological response to xenobiotics and natural compounds. Biomarkers such as lipid peroxidation, nitric oxide levels, and antioxidant enzyme activities (e.g., superoxide dismutase) are commonly used to evaluate cellular damage and defense mechanisms. In parallel, gene expression markers related to hepatotoxicity, apoptosis, endocrine regulation, cardiotoxicity, and developmental signaling provide deeper mechanistic insight into potential toxic or protective effects.

In parallel with increasing interest in natural products, there is a strong ethical and scientific imperative to refine preclinical safety assessment. The “3R” principle (replacement, reduction, and refinement), introduced by Russell and Burch in 1959, aims to minimize animal use and suffering while ensuring scientifically valid research [11]. The concept of responsibility is now also recognized as an additional guiding principle. In this framework, zebrafish (*Danio rerio*) embryos and larvae have emerged as valuable alternative models for early-stage screening, with Organisation for Economic Co-operation and Development (OECD) guidelines recognizing zebrafish embryos for systemic toxicity evaluation. Following initial cell-based screening, zebrafish embryos and larvae have been proposed as a second preclinical stage for toxicity detection and validation of potential therapeutic candidates [12].

Accordingly, the aim of this study was to investigate the biocompatibility of a newly developed herbal formulation that combines *Rhus coriaria* extract as an adjunct to propolis, using a comprehensive assessment of biochemical parameters and gene expression in zebrafish embryos.

## 2. Materials and Methods

The patent application for the sumac + propolis oral formulation containing *Rhus coriaria* extract was submitted to the Turkish Patent Institute on 21 September 2021, under application number 2021/014763. Intellectual property rights for this invention have been claimed by our university (Bursa Uludağ University) under case number 2021-30/12.08.2021. All plant-derived components and propolis were prepared and used as aqueous extracts. The contents of the test groups are provided in Table 1. Test concentrations were determined through preliminary range-finding toxicity assays. Zebrafish embryos were exposed to serial dilutions of each formulation. The highest concentrations that did not induce significant mortality or morphological abnormalities by 72 h post fertilization (hpf) were selected for further analysis. The propolis-only formulation was applied at 5%, and the Sumac–Propolis combination was applied at 10% *Rhus coriaria* plus 5% propolis. 3,4-Dichloroaniline (DCA) has been used as the Positive Control and Sumac + Propolis and Propolis groups were not exposed to DCA.

### 2.1. Chemical Characterization of the Herbal Formulation

Antioxidant activity was evaluated using the Cupric Ion Reducing Antioxidant Capacity (CUPRAC) and 2,2-diphenyl-1-picrylhydrazyl (DPPH) assays. Total phenolic content determination and dry matter analysis were performed using standard laboratory procedures. The phenolic compounds of propolis and sumac–propolis extracts were determined using high-performance liquid chromatography coupled with a diode array detector (HPLC-DAD) according to the method described by Eyüboğlu et al. (2025) [13]. Analyses were performed using an Agilent 1200 Series HPLC-DAD system equipped with a diode array detector, binary pump, autosampler, and vacuum degasser.

Chromatographic separation was achieved on an XBridge^®^ C18 column (4.6 × 250 mm, 3.5 μm). The mobile phase consisted of 1% formic acid in water (solvent A) and acetonitrile (solvent B). The flow rate was maintained at 0.5 mL min^−1^, and the injection volume was 20 μL. The total run time was 36 min, employing the following gradient program: 0–10 min, 13% B; 10–20 min, 4.5% B; 20–25 min, 7% B; and 25–35 min, 1% B.

Identification and quantification of phenolic compounds were carried out by comparing retention times and UV spectra with those of authentic standards. The concentrations of phenolic compounds in the extracts were expressed as mg/L of sample.

### 2.2. Maintenance of Zebrafish 

In this study, zebrafish embryos obtained by the natural spawning of AB/AB strain zebrafish were used [14]. The zebrafish were maintained in an aquarium rack system (Zebtec, Tecniplast, Buguggiate, Varese, Italy) at 28 ± 1 °C, under disease-free conditions with a 14/10 h light/dark cycle. They were fed twice daily with commercial flake fish food and live artemia. During the experiments, reverse osmosis water supplemented with 0.018 mg L^−1^ Instant Ocean™ salt was used. Fertilized embryos collected after natural spawning were staged according to their development and morphological criteria.

### 2.3. Embryo Exposure

Zebrafish embryos were exposed to 3,4-dichloroaniline (DCA group) as the positive control, the oral formulation without sumac (propolis group), and the oral formulation with sumac (sumac + propolis group) in well plates for 72 hpf. Embryo medium was used as the blank control. Their developmental stages were monitored and recorded every 24 h. To achieve an adequate number for RT–PCR and biochemical analyses, a total of six biological replicates of pools of zebrafish embryos, with 50 embryos/pool, were prepared.

Fresh solutions were substituted with the exposure solution every day. Using a stereomicroscope (Zeiss Discovery V8, Germany), developmental parameters were examined every day, and deformities were noted and photographed. As previously mentioned, the development indicators—yolk sac, anal pore, pectoral fin, and swim bladder—were utilized for embryo staging [15].

### 2.4. Evaluation of Biochemical Parameters

Lipid peroxidation, nitric oxide levels, acetylcholinesterase activity, and superoxide dismutase activity were evaluated as the biochemical parameters. These measurements were assessed to determine the potential effects of the oral formulation on oxidant–antioxidant status and acetylcholinesterase activity.

### 2.5. Embryo Homogenization for the Biochemical Parameters

Biochemical analyses were carried out on whole-embryo homogenates. Three biological replicates of pools of zebrafish embryos (50 embryos/pool) for each group were prepared to attain a sufficient amount for biochemical parameters. Following exposure, the embryos were sacrificed and homogenized using iron beads and a homogenizer to assess oxidant–antioxidant parameters. Prior to this procedure, the embryos were anesthetized using Tricaine (Sigma A5040, Darmstadt, Germany). The Tricaine solution was prepared by mixing 400 mg of Tricaine, 97.9 mL of distilled water, and 2.1 mL of 1 M Tris, adjusting the pH to 7. The prepared homogenates containing embryos were stored at −20 °C until the day of analysis.

### 2.6. Assessment of Biochemical Parameters

The lipid peroxidation (LPO) product malondialdehyde (MDA) reacts with thiobarbituric acid (TBA), forming a pinkish color. The absorbance of this color was measured spectrophotometrically to assess the level of lipid peroxidation. The butanol phase was used to measure the absorbance at 532 nm against the blank. The results were calculated using the extinction coefficient for MDA, 1.56 × 10^5^ M^−1^ cm^−1^, and the results are expressed as nmol MDA/mg protein [15].

Nitric oxide levels were determined by reducing nitrate to nitrite using vanadium (III) chloride. The nitrite reacts with sulfanilamide in an acidic environment to form a diazonium compound, which is then measured spectrophotometrically at 540 nm against the blank. The results were presented as nmol/mg P using the extinction coefficient of 53,000 M^−1^ cm^−1^ [16].

The Ellman (1961) [17] method was utilized to determine the level of Acetylcholinesterase AChE activity present in the supernatants. Using 5,50-dithiobis (2-nitro-benzoic acid), AChE in the homogenate creates thiocholine by generating a yellow hue. The yellow product’s color intensity was correlated with the sample’s enzyme activity and evaluated at 412 nm utilizing a spectrophotometer and the results are expressed as U/mg protein.

Superoxide Dismutase SOD activity was measured by its ability to increase the photooxidation rate of o-dianisidine in the presence of riboflavin. The absorbance of the colored product formed was measured at 460 nm using a spectrophotometer by determining net absorbances at 0 and 8 min. The SOD activity in U/mg protein per minute was calculated using the standard curve.

### 2.7. Reverse Transcription and Quantitative Real-Time PCR for Gene Expression Analysis

RNeasy Mini Kit and QIAcube device (Qiagen, Hilden, Germany) were used to isolate RNA from the embryos following the manufacturer’s instructions. Three biological replicates of pools of zebrafish embryos (50 embryos/pool) for each group were prepared to attain a sufficient RNA pool. RT2 Profiler PCR Arrays (Qiagen, Hilden, Germany) were used to synthesize the single-stranded cDNA from total RNA, and PCR analyses were performed using the DNA Master SYBR Green kit (Qiagen, Hilden, Germany).

Table 2 presents the biological mechanisms associated with the examined genes, highlighting their roles as indicators of specific physiological and pathological processes. The expression levels of *fabp10a*, *cyp1a*, *hsp70*, *bax*, *bcl2*, *vtg*, *gfap*, *erg*, and *igf2* were quantified using quantitative reverse transcription PCR (qRT–PCR) with the Rotor-Gene system (Qiagen, Hilden, Germany). The list of the forward and reverse primers is given in Table 3. Relative transcription levels were measured using the DDCT method, with values normalized with ß-actin, the housekeeping gene.

### 2.8. Statistical Analysis

The effects of the oral formulation on zebrafish embryos were evaluated using one-way ANOVA, following data normality analysis using the Shapiro–Wilk test. Biochemical parameters and gene expression were considered dependent variables. A post hoc test appropriate for the distribution was used for multiple comparisons. Statistical analysis was performed using GraphPad Prism 9. The results were presented as mean ± standard deviation, and a significance level of *p* < 0.05 was considered.

## 3. Results

The antioxidant and phenolic characteristics of the formulation are presented in Table 4.

The phenolic composition of the propolis and sumac–propolis extracts revealed significant qualitative and quantitative differences. Sumac–propolis formulation exhibited a markedly higher total abundance of phenolic compounds than propolis. Among the identified compounds, gallic acid (538.77 ± 9.62 mg/L) was the predominant phenolic compound in sumac–propolis formulation and was not detected in propolis. Similarly, epigallocatechin gallate (95.84 ± 4.79 mg/L), epicatechin gallate (58.92 ± 0.97 mg/L), and ellagic acid (49.34 ± 0.01 mg/L) were exclusively detected in sumac–propolis formulation, contributing substantially to its enhanced phenolic content and antioxidant potential (Table 5).

**Figure 1:**
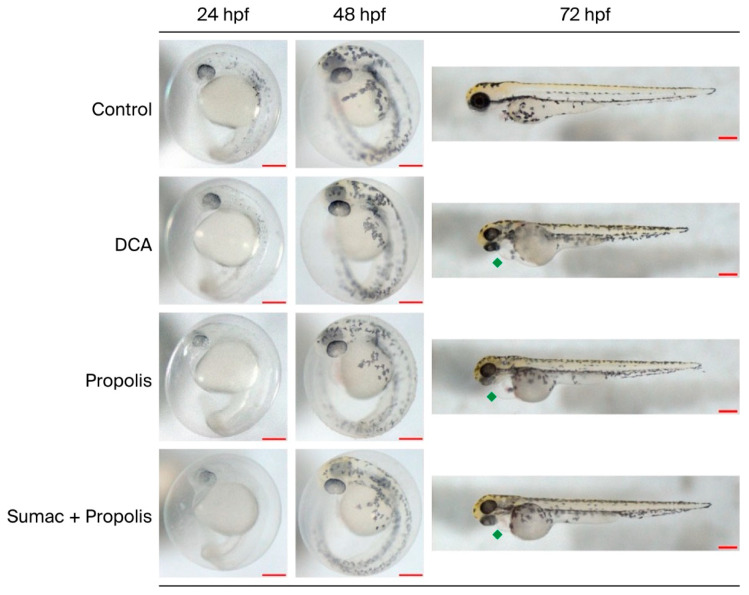
Microscopic images showing the morphological features of embryos in the control and exposure groups. Green arrowhead: indicates pericardial edema. Scale bar: 250 µm.

Figure 1 shows the representative images of the zebrafish embryos at 24, 48, and 72 hpf for the control, 3,4-dichloroaniline (DCA), the oral formulation without sumac (propolis group), and the oral formulation with sumac (sumac group). In the case of exposure to DCA, pericardial edema was used as a positive control. Pericardial edema was also observed in the formulation without sumac, but fewer embryos were found, and it was less severe than the formulation with sumac. In the sumac-containing formulation group, pericardial edema was observed in fewer embryos and to a lesser degree.

### 3.1. Biochemical Parameters

**Figure 2:**
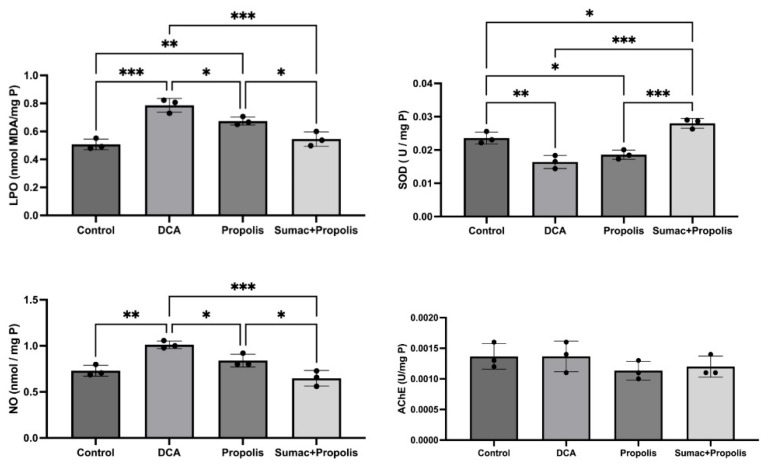
Comparison of lipid peroxidation (LPO), nitric oxide (NO), superoxide dismutase (SOD), and acetylcholinesterase (AChE) activities between groups. Data are expressed as the mean ± SD from three independent experiments (*n* = 3, three biological replicates for each group, 50 embryos/pool). *** *p* < 0.001; ** *p* < 0.01; * *p* < 0.05.

Comparisons of lipid peroxidation (LPO), superoxide dismutase (SOD), nitric oxide (NO), and acetylcholinesterase (AChE) activities among the experimental groups (Control, DCA, Propolis, and Sumac + Propolis) are shown in Figure 2. These results provide insight into the oxidative stress and neurotoxicity in the embryos exposed to DCA, the oral formulation without sumac, and the oral formulation with sumac.

Lipid peroxidation (LPO) levels were significantly higher in the DCA group compared with the control group (*p* < 0.001). LPO levels also showed a statistically significant increase in the formulation without sumac compared with the control group (*p* < 0.01). In the formulation with sumac, however, LPO levels were not different from the control group and were significantly lower than those in the DCA and sumac-free formulation groups (*p* < 0.05).

Nitric oxide (NO) levels were significantly higher in the DCA group compared with the control group (*p* < 0.01). NO levels in both the formulation without sumac and the formulation with sumac were not different from the control group and were significantly lower than those in the DCA group (*p* < 0.05).

Superoxide dismutase (SOD) activities were significantly reduced in the DCA group compared with the control group (*p* < 0.01). SOD activity was also significantly lower in the formulation without sumac compared with the control group (*p* < 0.05). In the formulation with sumac, SOD activity was significantly higher than the control, DCA, and sumac-free formulation groups (*p* < 0.05, *p* < 0.001, and *p* < 0.001, respectively).

When comparing the acetylcholinesterase (AChE) activities as part of the overall biocompatibility evaluation for detecting neurotoxicity, no statistically significant differences were found between the groups.

### 3.2. Gene Expression Analysis

**Figure 3:**
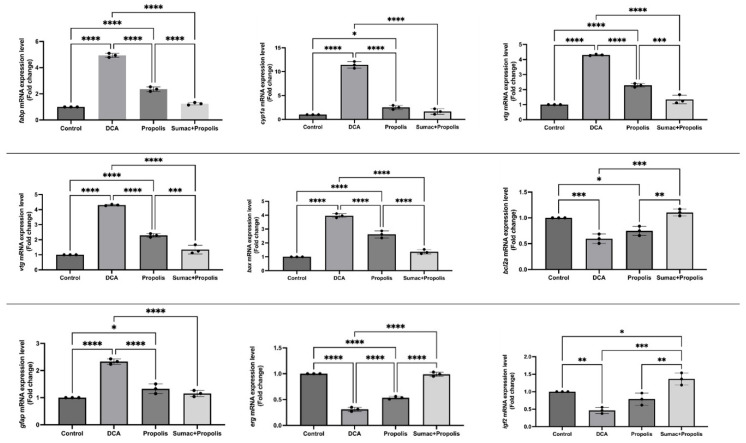
Comparison of gene expression levels between experimental groups. (RT–PCR results were normalized to beta-actin, the housekeeping gene, and expressed as the change from their respective controls. The average values were obtained from three experiments (*n* = 3, three biological replicates for each group, 50 embryos/pool). Data presented are mean ± SD. *** *p* < 0.001; ** *p* < 0.01; * *p* < 0.05; **** *p* < 0.0001.).

Relative mRNA expression levels of genes associated with hepatotoxicity (fabp10a), xenobiotic biotransformation (cyp1a), apoptosis and antioxidant/DNA repair mechanisms (bax, bcl2), steroid pathway (vtg), neurotoxicity (gfap), cardiotoxicity (erg), and growth/development (igf2) in zebrafish embryos across experimental groups (Control, DCA, Propolis, and Sumac + Propolis) are shown in Figure 3. Gene expression levels were quantified using qRT-PCR and normalized to the control group (fold change).

Considering that *fabp10a* is used as a biomarker for hepatotoxicity in zebrafish, in our study, *fabp* expression was significantly increased in the DCA group compared with the control group (*p* < 0.0001). *fabp* expression was also increased in the sumac-free formulation group compared with the control group (*p* < 0.0001). In the formulation with sumac, however, the *fabp* expression was not different from the control group and was significantly lower than in the DCA and sumac-free formulation groups (*p* < 0.0001).

The expression of genes involved in biotransformation, such as *cyp1a*, is induced by the interaction of aromatic compounds with the cytosolic aryl hydrocarbon receptor (AhR). Several examples of these compounds, including PAHs, pesticides, pharmaceuticals, PCBs, PBDEs, PCDDs, and triclosan, have been shown to activate AhR-mediated responses in zebra fish larvae. In our study, *cyp1a* expression was significantly increased in the DCA group compared with the control group (*p* < 0.0001). *cyp1a* expression was also higher in the sumac-free formulation compared with the control group (*p* < 0.05). In the formulation with sumac, however, *cyp1a* expression was not different from the control group and was significantly lower than in the DCA group (*p* < 0.0001).

*vtg* gene expression increases in the presence of xenoestrogens. In our study, DCA exposure significantly increased *vtg* expression (*p* < 0.0001). Although *vtg* expression increased in the propolis group, no significant difference in *vtg* expression was observed in the group treated with the formulation containing sumac. Moreover, *vtg* expression in the sumac-containing formulation group was significantly lower than the DCA and propolis group (*p* < 0.0001 and *p* < 0.001, respectively).

Moreover, DCA exposure significantly upregulated pro-apoptotic *bax* expression (*p* < 0.0001) while downregulating anti-apoptotic *bcl2* expression (*p* < 0.001). However, in the formulation containing sumac, *bax* and *bcl2* expressions did not exhibit statistically significant differences compared with the control group.

*gfap* expression, an indicator of neurotoxicity, increased significantly in the DCA group. Although there was a significant increase in *gfap* expression in the propolis group, no significant change was observed in the sumac + propolis group.

In our study, DCA exposure significantly reduced *erg* expression (*p* < 0.0001). Although the propolis group also reduced *erg* expression, albeit to a lower extent (*p* < 0.0001), no statistically significant difference in *erg* expression was observed in the group treated with the sumac-containing formulation. Moreover, erg expression in the sumac-containing formulation group was statistically significantly higher than in the DCA and without sumac groups (*p* < 0.0001).

In our study, DCA exposure significantly reduced *igf2* expression (*p* < 0.01). In the sumac-containing formulation group, *igf2* expression was significantly increased compared with the control (*p* < 0.05), and it was statistically significantly higher than in the DCA and propolis groups (*p* < 0.001 and *p* < 0.01, respectively).

## 4. Discussion

Zebrafish embryos are widely regarded as a robust and reproducible alternative vertebrate model in biomedical research, offering practical advantages such as high fecundity, optical transparency, rapid organogenesis, ease of laboratory maintenance, and access to advanced genetic tools [25]. Their developmental biology and sensitivity to chemical exposures have also made zebrafish embryos a preferred system in environmental toxicology and in early-stage screening of bioactive compounds. Accordingly, the Organisation for Economic Co-operation and Development (OECD) has established internationally accepted procedures for zebrafish embryo toxicity testing, including OECD Test Guideline 236 for the Fish Embryo Acute Toxicity (FET) test [26]. In embryotoxicity assessment, exposure ranges are commonly selected based on early developmental stages (e.g., 24 h post-fertilization (hpf)) to determine concentration-dependent effects on survival and development.

Propolis represents a chemically complex bee-derived matrix rich in phenolic compounds and other constituents that underpin its biological activity but may also contribute to variability in tolerability across formulations. Therefore, alongside efficacy-oriented studies, systematic safety assessment of propolis-containing preparations is increasingly warranted. In this study, we evaluated the safety of two oral herbal formulations containing predominantly propolis and *Rhus coriaria* using a subacute toxicity testing approach in zebrafish embryos in accordance with OECD 236. The formulation strategy aimed to maintain the beneficial bioactivity associated with propolis while improving biocompatibility by incorporating *Rhus coriaria* and reducing propolis content.

The biological activities of sumac are largely attributed to its diverse phenolic composition, which is dominated by hydrolysable tannins, flavonoids, anthocyanins, and phenolic acids. Among these compounds, gallic acid and its derivatives are considered key contributors to the antioxidant capacity of sumac. Gallic acid exhibits strong radical scavenging and reducing activities owing to its multiple hydroxyl groups and has also been associated with antimicrobial effects against various pathogenic microorganisms [27,28,29].

The synergistic interaction among gallic acid derivatives, flavonoids, anthocyanins, and tannins is believed to be responsible for the broad spectrum of biological activities observed in sumac extracts. These include antioxidant, anti-inflammatory, antimicrobial, anti-biofilm, gastroprotective [30], and neuroprotective effects. Consequently, the phenolic-rich composition of sumac supports its potential use in functional foods, nutraceuticals, pharmaceuticals, and cosmetic formulations [31,32,33].

Oxidative-stress-related endpoints are particularly relevant for assessing the safety and biological plausibility of natural-product formulations. Lipid peroxidation is a key contributor to cellular damage and serves as an established biomarker of oxidative stress [34]. *Rhus coriaria* has been reported to exert antioxidant and anti-inflammatory activities in multiple experimental settings [35], and in our study, lipid peroxidation levels in the sumac-containing formulation were significantly lower than those observed in the DCA and propolis groups, supporting an improved redox profile of the combined preparation.

Nitric oxide (NO) signaling is implicated in inflammation and carcinogenesis through its effects on angiogenesis, tumor progression, and metastasis [36]. Prior evidence suggests that *Rhus coriaria* can modulate inflammatory and NO-related pathways, including suppression of NFκB/STAT3 signaling [37]. Consistent with this mechanistic rationale, our findings indicate that *Rhus coriaria* not only exerted a favorable influence on NO-related outcomes but also enhanced the profile of the propolis preparation, suggesting a more chemoprotective pattern in the combined formulation.

The antioxidant capacity of *Rhus coriaria* has also been linked to the modulation of enzymatic defenses. In models of oxidative stress, *Rhus coriaria* extract has been reported to support antioxidative responses by upregulating enzymes such as superoxide dismutase and catalase [38]. In our study, the SOD activity was significantly higher in the *Rhus coriaria* formulation compared with the control, DCA, and propolis-based formulation groups, aligning with previous observations of superoxide radical scavenging and xanthine oxidase inhibition by *Rhus coriaria* [39].

Beyond systemic oxidative stress markers, hepatotoxicity-related outcomes are important for oral formulations intended for patients with compromised mucosal integrity. Preclinical evidence has suggested hepatoprotective effects of *Rhus coriaria* in rodent models of chemical liver injury [40], and protective effects against oxidative stress in hepatocytes have also been reported [41]. In line with this literature, hepatotoxicity-related effects in our study, assessed via *fabp* gene expression, were significantly lower in the *Rhus coriaria* formulation compared with DCA and propolis formulations, supporting an improved safety profile.

Given that phytochemicals may affect endocrine-related endpoints in fish, reproductive-axis genes provide an additional safety lens. Phytochemicals can influence estradiol signaling by altering aromatase activity or interacting with estrogen receptors, potentially altering vitellogenin (*vtg*) synthesis [42]. In our gene expression analyses, the sumac-containing formulation yielded *cyp1* and *vtg* expression patterns closer to the control and distinct from DCA, indicating that the formulation improved the outcomes observed with the propolis preparation.

Apoptosis-related markers also provide mechanistic insight into toxicant-induced injury. DNA damage and cellular stress can activate p53-regulated pathways, increasing pro-apoptotic *bax* and decreasing anti-apoptotic bcl2, with the bcl2/bax balance serving as a sensitive indicator of apoptotic susceptibility [43]. In the present study, DCA exposure increased bax and decreased *bcl2* expression, whereas the addition of *Rhus coriaria* mitigated these alterations, resulting in expression levels not significantly different from those of the control group.

Cardiotoxicity is a critical consideration in zebrafish embryo screening. The *erg* gene encodes a potassium channel subunit relevant to cardiac electrophysiology and is widely used as a biomarker of cardiac toxicity in zebrafish [23,44]. Our results showed that DCA significantly reduced *erg* expression compared with the propolis and sumac formulation groups. Although *erg* expression did not differ significantly between the *Rhus coriaria* and propolis groups, it was significantly higher in the *Rhus coriaria* formulation than in the DCA and propolis groups, indicating a more favorable cardiac-related profile in the combined formulation.

Finally, developmental growth signaling can be captured through GH/IGF-axis markers. The *igf2* gene, expressed in the liver and involved in growth and development [45], was significantly reduced by DCA exposure in our RT–PCR analysis. In contrast, the sumac-containing formulation significantly increased *igf2* expression compared with the control and with the DCA or propolis groups, suggesting improved developmental tolerance.

Collectively, while the beneficial effects of propolis on oral mucositis have been reported [46,47], our findings support the need for careful safety-focused research into propolis-based products. In this context, the incorporation of Rhus coriaria appears to modulate oxidative stress, inflammatory signaling, and gene expression profiles in a manner that enhances overall biocompatibility. Within this framework, reducing the propolis proportion and incorporating *Rhus coriaria* as a complementary botanical component resulted in measurable improvements across biochemical markers and gene expression endpoints in zebrafish embryos.

This study has several limitations that should be acknowledged. Zebrafish embryos possess a functional innate immune system that is highly conserved with mammals and plays a key role in early inflammatory responses to xenobiotic exposure. Innate immune parameters in zebrafish larvae—such as neutrophil migration, oxidative burst, and inflammatory cytokine expression—have been shown to correlate well with those observed in humans [48]. Therefore, although zebrafish embryos provide a well-established and sensitive in vivo model for early toxicity and biocompatibility assessment, the results may not fully reflect the complexity of mammalian systems. Second, while the experimental design allowed a controlled comparison between formulations differing only in the presence of *Rhus coriaria*, the multi-component nature of the formulation may still introduce potential interactions among constituents that could influence the overall biological response. Pericardial edema and other morphological abnormalities were assessed qualitatively during the experiments. While these observations provided initial insight into the developmental effects of the formulations, a quantitative scoring system was not employed. This represents a limitation of the study, and future investigations should incorporate quantitative morphological assessments to complement biochemical and molecular findings. In addition, the chemical characterization of the formulation was limited to total phenolic content analysis, and more detailed profiling using advanced analytical techniques such as HPLC or LC–MS would further strengthen the interpretation of the findings. Furthermore, the study focused on short-term exposure and selected biochemical and gene expression markers; therefore, long-term effects and additional endpoints such as histopathological evaluation remain to be investigated. Finally, the results suggest improved biocompatibility with the inclusion of *Rhus coriaria*; further validation using mammalian cell models and clinical studies is necessary to confirm its translational relevance.

## 5. Conclusions

The present study demonstrated that incorporation of *Rhus coriaria* extract into a propolis-based oral formulation improved the biocompatibility profile in zebrafish embryos compared with a propolis-only preparation. The combined formulation mitigated oxidative stress, normalized key biochemical parameters, and favorably modulated gene expression patterns associated with hepatotoxicity, apoptosis, neurotoxicity, cardiotoxicity, and developmental signaling. Importantly, these effects were achieved without altering acetylcholinesterase activity, indicating preserved neurofunctional integrity. Collectively, the findings suggest that *Rhus coriaria* may act as a supportive phytochemical component that enhances the safety profile of propolis-containing formulations while maintaining their biological potential. This formulation strategy warrants further investigation through advanced preclinical models and clinical studies to explore its translational relevance for oral mucositis-oriented therapeutic applications.

## Figures and Tables

**Table 1:** Study groups.

Control	DCA (Positive Control)	Sumac + Propolis	Propolis
Embryo Medium 5 mM NaCl 0.17 mM KCl 0.33 mM CaCl_2_ 0.33 mM MgSO_4_	3,4-Dichloroaniline	10% *Rhus coriaria* * 5% pure propolis ** Hyaluronic acid *** Black mulberry extract **** German chamomile *▲ Sage **▲ Xylitol ***▲ Peppermint flavor ****▲ Mono propylene glycol Glycerin Sucralose Purified water	5% Pure propolis ** Hyaluronic acid *** Black mulberry extract **** German chamomile *▲ Sage **▲ Xylitol ***▲ Peppermint flavor ****▲ Mono propylene glycol Glycerin Sucralose Purified water

* Harvested in the Gaziantep region, Turkey; aqueous extract. ** CAS: 85665-41-4, Talya Herbal^®^, Antalya, Türkiye, Antalya German chamomile. *** ≥95% purity, Batch No: HA2020111016X, Xinjiang Fufeng Biotechnologies^®^, China. **** CAS No: 90064-11-2; SRS Aromatics^®^, Bury St. Edmunds, UK. *▲ CAS No: 84649-86-5, Talya Herbal^®^, Antalya, Türkiye **▲ CAS No: 85085-68-3, Talya Herbal^®^, Antalya, Türkiye ***▲ Batch No: 120032602, Hylen^®^, China. ****▲ Product Code: FM052954, Aromsa^®^, Istanbul, Türkiye.

**Table 2:** Functional roles of examined genes and supporting literature.

Gene Expression	Parameter	Related Literature
fabp10a	Hepatotoxicity	Nguyen et al. (2017) [18]
cyp1a	Xenobiotic biotransformation	Casatta et al. (2017) [19]
bax, bcl2	Antioxidant defense mechanisms, apoptotic, DNA repair mechanisms	Wiebe et al. (2010) [20]
vtg	Steroid pathway	Vigano et al. (2020) [21]
gfap	Neurotoxicity	McGrath & Li (2008) [22]
erg	Cardiotoxicity	Vijayaraj et al. (2012) [23]
igf2	Growth/development	Hartnett et al. (2010) [24]

**Table 3:** List of forward and reverse primers used in the study.

	Forward	Reverse
***erg***	GTGGGTTATGACGCTGTCAG	CTAACTGCGCTCTCTGCTC
***gfap***	TGCGAACTGTTGAGACCCGT	TCTTCTGCAGCCAAGCCAGT
***vtg***	CAAGAGGCTGGAGCTCAGGG	CTCTCCTGGCAGTGGCTCAG
***cyp1a***	TCCAACCTGCAAGTGTCCGA	AGTGGTGTGCGATCCTTCCC
***igf2a***	TACTGTGCCAAGCCGGTGAA	GGGCCAACAGAATGGATGGG
***fabp10a***	CACCATGGACGGCAAGAAGC	GTTCCTCCGACTGTCAGCGT
***bcl2***	TGGAGGTTGGGATGCCTTCG	ATTGGCATGGAGACCGCAGA

**Table 4:** Antioxidant and phenolic characterization of sumac + propolis formulation.

Analysis	Result
CUPRAC antioxidant activity	9.74 ± 0.10 mmol TE */100 mL
DPPH antioxidant activity	7.60 ± 1.16 mmol TE/100 mL
Total phenolic content	15.96 ± 0.20 mg GAE **/100 mL
Dry matter content	23.98 ± 0.15%

* Trolox Equivalent; ** Gallic Acid Equivalent.

**Table 5:** Phenolic compound concentrations in propolis and sumac–propolis formulations (mg/L).

Phenolic Compounds	Propolis (mg/L)	Sumac–Propolis (mg/L)
Protocatechuic acid	4.63 ± 0.34	nd
*p*-Hydroxybenzoic acid	3.86 ± 0.01	nd
Vanillic acid	5.50 ± 0.13	nd
Chrysin	12.84 ± 0.13	13.55 ± 0.35
Gallic acid	nd	538.77 ± 9.62
Epigallocatechin gallate	nd	95.84 ± 4.79
Epicatechin gallate	nd	58.92 ± 0.97
Hesperetin	60.64 ± 0.90	76.03 ± 0.13
Eriodictyol	49.01 ± 0.06	63.25 ± 0.75
trans-Cinnamic acid	27.79 ± 0.23	27.78 ± 0.06
Pinocembrin	24.92 ± 0.19	29.63 ± 0.04
*p*-Coumaric acid	1.70 ± 0.04	3.96 ± 0.02
Ferulic acid	38.00 ± 0.14	51.55 ± 0.09
Rosmarinic acid	2.82 ± 0.08	nd
Resveratrol	23.59 ± 0.24	26.02 ± 0.07
Ellagic acid	nd	49.34 ± 0.01
Galangin	15.72 ± 0.55	20.93 ± 0.32

mean ± standard deviation; nd: not detected.

## Data Availability

Data will be available on reasonable request.
